# Coverage of Child Disability Detection, Management, and Rehabilitation Health Services in Central Uganda

**DOI:** 10.24248/eahrj.v8i2.778

**Published:** 2024-06-26

**Authors:** Edith Akankwasa, Willy Kamya, Moses Sendijja, Janet Mudoola, Mathias Lwenge, Robert Anguyo DDM Onzima, Simon-Peter Katongole

**Affiliations:** a Mildmay Institute of Health Sciences; b Department of International Public Health, Liverpool School of Tropical Medicine; c Gudie University Project

## Abstract

**Background:**

Child disability is a significant public health concern which impacts 1 in 20 children. Children with disabilities (CwDs) encounter deprivation of rights, biases in society, and a lack of access to necessary services, all of which are exacerbated by structural obstacles. This study assessed the coverage of child disability prevention, management and rehabilitation services in four districts of Central Uganda after two years of interventions to improve these services.

**Methods:**

The Lot Quality Assurance Sampling (LQAS) rapid health facility assessment method was employed to assess coverage of services based on sixteen indicators. The indicators were set based on constructs of: provision of disability-related services to CwDs; use of rehabilitation services; readiness of the health facilities (HFs) to provide basic disability management and rehabilitation services; community structures for linkage to disability management and rehabilitation services; psychosocial support provision; and upholding and protecting the rights of CwDs. A district-level decision rule was set based on 80% coverage target.

**Results:**

Despite the interventions, the services have yet to provide the desired level of benefit to CwDs and their caregivers. Out of the sixteen indicators for healthcare service coverage for CwDs, only three attained the 80% coverage target.

**Conclusion:**

The findings suggest that greater focus by health service planners and project implementers is still needed, especially at the community and health facility levels to enhance the prevention, management and rehabilitation of CwDs. Psychosocial health services for caregivers of CwDs need special attention in order to achieve better service approaches.

## BACKGROUND

Child disability has become one of the leading public health concerns globally with one in every 20 children having a moderate or severe form of disability.^1^ Disabilities in children include visual, hearing, and physical disabilities, as well as mental health challenges such as cognitive impairment, intellectual disability, communication, and mental illness.^2^ Most of the Children with Disabilities (CwDs) are confined into isolation and deprived of various rights, including the right to education and access to health services such as mental health services ^3^. In addition, the CwDs face prejudices and discriminations in their societies due to the negative cultural attitudes some communities have towards them ^4^. In Africa, the proportion of children aged 14 years and below with any form of disability is reported to be 6·4%.^5^ In Uganda, around 4% of the children below five years old have a form of disability.^6^ On the other hand, 66.2 of every 10,000 live births to neonates have a birth defect.^7^

Despite the need to ensure that CwDs receive the much-needed services to improve their health and reach their potential in life, public health services in most sub-Saharan countries rarely target them.^8^. Some of them are deprived of food, making them prone to nutrition-related problems thus affecting their physical growth due to the resulting undernutrition and other nutritional deficiencies.^2,9,10^ Even when services are available to improve the health of CwDs, some parents hide the children from such services.^11^ Besides, even when CwDs grow up, they often underestimate themselves in their communities further complicating efforts to reach out to them.^12^ This is exacerbated by the health providers and community members who fail to realize the challenges CwDs and their caregivers face so that they may accord them the necessary help that they need.^13^ Weak coordination, advocacy and response by governments, civil society and communities also exacerbate the challenge of providing accessible services for CwDs.^8^

As part of the efforts to improve the health of CwDs, Mildmay Institute of Health Sciences (MISH) collaborated with several organizations and rehabilitation centres to implement child disability prevention, detection, management, and rehabilitation (CDR) services in the districts of Mubende, Kassanda, Mityana, and Luwero located in Central Uganda. Further investigation into the project which is partially linked to this study and explains the strategies for safe motherhood, birth defects preventions, detection and management is detailed in a separate publication.^14^ This study on the other hand discusses the identification, treatment, rehabilitation, and prevention of disabilities during the initial five years of a child's life.

## MATERIALS AND METHODS

### Study Design

A cross-sectional research design was used to collect data for this study. The intervention outcomes are examined using quantitative methods. This entailed reviewing District Health Information Software 2 (DHIS2) data from HFs to extract information on specific indicators of service delivery to CwDs. Other methods included health-care facility-based surveys to determine proportion of under-five CwDs who accessed CDR services in comparison with the project target.

### Study Sites

The research was carried out in Mubende, Mityana, Kassanda, and Luwero districts. The information was gathered between the months of May and August of 2021. The interventions were carried out in 61 HFs which constituted the sampling frame (Mityana: 24; Mubende: 10; Kassanda: 13; and Luwero: 13). In this study, the supervision area was formed by a district, with all the four districts forming a catchment area. Data on the indicators of interest were collected from each sampled health facility (HF), either at the facility or from households in the area served by the facility. Data on indicators having sources within the HF were gathered from HF while those with no HF-based source were collected from household survey.

For the sampling of HFs in the study districts, the hypergeometric-based rapid health facility assessment (r-HFA) was used, while binomial Lot Quality Assurance Sampling (LQAS) was used to sample communities (interview locations) from the catchment areas of the sampled HFs. LQAS is a well-known classification method that was introduced into public health in the 1980s and has since been used to ensure the quality of health programs in developing countries.^15^ It has primarily been used for assessing the success of a local health team in supervision areas (SAs) in adequately covering the population with an intervention, and assessing health worker performance in terms of quality of care and the status of equipment and supplies at HFs.^16^ LQAS's power stems from the fact that it is used to classify supervision areas based on whether or not they have met a performance target.^17^ In this study, this performance target corresponds to the decision rule (DR). The LQAS approach requires far less data than is typically required to classify a coverage proportion in an area.^18^

### The Intervention

MIHS collaborated with several organizations and rehabilitation centres to implement CDR services in four districts^14^. In the community, the project boosted community-based rehabilitation services by training selected village health team members (VHTs) to improve their advocacy skills for demand creation towards child disability services. In addition, the VHTs were trained on how to detect disabilities in children and to link CwDs to appropriate disability management and rehabilitation services at HFs where they are provided. In addition, health workers in the districts underwent training while the HFs were equipped with some tools to provide services related to safe motherhood, prevention, and early detection of birth defects. The training focused on identifying and preventing birth defects, offering prenatal care to address these issues where possible, and ensuring proper management for infants suspected of having congenital challenges. The project included interventions for children identified with disabilities later in life or soon after birth. It aimed to address child disabilities comprehensively from prebirth through the first five years, believing early interventions would yield better outcomes, enhancing the quality of life for CwDs. Further investigation into this specific aspect of the project which is partially linked to this study and explains the strategies for safe motherhood, birth defects preventions, detection and management is detailed in a separate publication^14^. The present study on the other hand discusses the identification, treatment, rehabilitation, and prevention of disabilities during the initial five years of a child's life.

The project targeted CwDs aged 0-59 months because it was determined that better rehabilitation results could be achieved if disabilities were detected early and addressed before the children started school, and that better rehabilitation and corrective outcomes could be achieved than when rehabilitation and disability correction were deemed too late to achieve the best results.

The project identified and trained community-based organizations (CBOs), opinion leaders, and other community resource people, such as VHTs, to care for disabled children. Sub-County and district community development officers, councilors of persons with disabilities, district and sub-county women councilors, and selected local council members were among the community leaders trained. The leaders mentioned above were also trained to advocate for and promote services for CwDs, as well as to track CwDs in the community in order to connect them to care.

The health professionals targeted for training and inclusion in the project included medical officers, clinical officers, midwives, occupational health therapists, and VHTs. Medical officers, clinical officers and Midwives from government and private non-profit health organizations were trained as part of the intervention to provide basic management and rehabilitation of childhood disabilities at HFs, as well as to transfer CwD to referral centers where complex rehabilitation of disabilities could be carried out. They were also taught and trained on how to provide antenatal, perinatal, and postnatal care, with the prevention and management of identified disabilities at the forefront of the training content. Four people were targeted at the hospital level (one medical officer, two midwives, and one occupational therapist), two people (a midwife and a clinical officer) from health centers III and IV, and one person (a midwife) from participating HFs. One VHT from each participating HF was trained to identify CwDs and refer them to the local HF, as well as to coordinate and support the psychosocial services of family support groups (FSGs) formed for CwD caregivers in their area. A midwife served as the project's steward at each of the HFs. The mode of delivery of the training included classroom lectures, practical demonstration and visits to model rehabilitation centres and HFs.

The CwD were referred to rehabilitation centers based on their disability type. Referral mechanisms were developed, including a referral directory with information such as the types of disabilities handled at the referral center, as well as the names of people, addresses, and phone numbers at the referral center. These were given to all healthcare professionals so that they would know where to refer a CwD patient. Midwives, VHTs, and community development officers used radio talk shows, community dialogue, and community outreach to advocate for CDR services and to reduce stigma and violence against disabled children. Health workers also distributed information, education, and communication materials to raise awareness of the services offered, identify additional CwD, and provide access to HFs that offer basic disability management services.

Other interventions included providing psychosocial support to caregivers of disabled children. This approach's interventions included mobilizing and training caregivers and family members of CwDs in business, financial literacy, and how to add value to the business of their choice, with the goal of strengthening the socioeconomic status of CwDs' families and removing some of the bottlenecks that prevent CwDs from accessing appropriate disability health services. Among the barriers addressed were food insecurity, a lack of funds to access health services, including transportation and facilitation at rehabilitation centers, boosting self-reliance skills, eliminating gender inequality, and exclusion from social and health services.

In addition, project implementers collaborated with communities to form ten FSGs comprised of CwDs' caregivers. These FSGs were given vocational training in order to improve their socioeconomic status and gain access to various government and non-government social services for vulnerable populations. The FSGs were given vocational training in areas such as craftsmanship, liquid soap production, book production, hairdressing, and handcrafts. The FSGs would select a vocational skill of interest, and then the project implementers, in collaboration with the districts, would assign an artisan with training skills in that specific skill to the group to train the members for a set period of time.

The FSGs were also meant to promote the togetherness of parents and caregivers of CwDs. The FSGs were introduced to the concept of village savings and loan associations (VSLAs); the caregivers of CwDs were encouraged to become members in the VSLA to enable them to access financial resources and other benefits that came with being members. Besides, CwDs' families that were venturing into agriculture were also trained in modern farming skills aimed at helping them add value to their agricultural produce as well as to improve on their marketing skills, sale of their products, and increase profits in order to meet their financial needs.

The following were the service pathways for the services provided in this project: once a child was suspected or identified by a VHT to have a disability or developmental challenges, the mother or caretaker of the child was referred to an area HF that had the capacity to screen CwDs. Children presenting to a HF for illness treatment, routine HF services such as immunization and growth monitoring, and newly conceived children would all be screened for disability. This covered the project's disability detection component. Those who were discovered to be disabled were managed for conditions that the HF could handle, while those that they couldn't handle were referred elsewhere, as previously explained. The children received rehabilitation services at the referral centers, while their caregivers received psychosocial support. When the children were discharged from the rehabilitation centers, they would be linked back to the HFs in their communities, and the caregivers would be linked to FSGs. The trained midwives continued to monitor the children on scheduled days at the HFs, while the VHTs did so in the communities.

The project coordinator and field coordinators at MIHS collaborated with the respective Assistant District Health Officers in charge of maternal and child health to coordinate, monitor, and supervise project implementation. Quarterly reports were regularly submitted to the field coordinator, which aided in keeping track of the project's progress. The project interventions were integrated into existing services as an addition to routine antenatal, perinatal, and postnatal care services, the physiotherapy and occupational therapy department, community-based services provided by the VHT, social services provided by community development officers, and other civil societies and organizations (CSO) providing child disability related services within the communities. MIHS funded the project activities with funds provided by a funder who preferred to remain confidential in any future research and project information dissemination. Three years after the interventions were implemented, an evaluation was carried out to assess the coverage of the child disability services indicators in the four intervention districts along the project cascade.

### Study Population

The population of the study were the HFs providing child disability detection, management and rehabilitation services in the four study districts as well as households in their catchment areas.

### Inclusion and Exclusion Criteria

HFs that had been participating in providing child disability detection, management and rehabilitation services with support of MIHS were included in this study. Caregivers or parents of CwDs aged 0 to 59 months who live in the catchment area of the HFs providing child disability detection, management and rehabilitation services with support of MIHS were included for the community household survey. The HFs that were not supported by MIHS and caregivers or parents of under-five CwDs who were visitors to catchment areas of the corresponding HFs were excluded. Those who did not consent to participate in the study were also excluded.

### Sample size and sampling of HFs

The upper threshold (‘‘pU’’) or satisfactory coverage, and the lower threshold (‘‘pL‘‘) or the unsatisfactory coverage of a CDR service were set at 80% and 50% respectively with the worst acceptable α and β errors each at 10% (i.e. 0.1)^14^. The alpha (α) error in this study was considered as the probability that in the analysis of performance, a district that had achieved satisfactory coverage for an indicator was adjudged as having not achieved the desired coverage.^19^ On the other hand, the beta (β) error is the probability that a district that had not achieved satisfactory coverage was adjudged as having achieved the desired coverage.^19^ The DR in this study is the minimum number of HFs out of the total sampled HFs in each district that demonstrated satisfactory coverage for a district to be flagged off as having performed well on a given indicator. This generated sample sizes (n) of 13 and 7 HFs for Mityana and Mubende respectively as well as 10 each for Kassanda and Luwero. However, since the minimum sample size that the hypergeometric model deals with is 8, the sample size for Mubende was increased to eight, resulting in a total sample size of 41 HFs.

Based on the parameters in Table 1 and the estimated sample sizes and their corresponding DRs, the actual α for Mityana district was 0.0303 while the rest of the districts had a 0 α error. The actual β errors for Mityana, Mubende, Luwero, and Kassanda districts were 0.0498, 0.000, 0.0350, and 0.0699 respectively (Table 1).

**Table 1.** Sample Size Estimation for HFs in the Districts

District	HC II	HC III	HC IV	Hospital	No. of eligible HFs	Sample size (n)	α error	β error	DR
Mityana	5	15	3	1	24	13	0.0303	0.0498	9
Mubende	2	7	0	1	10	8	0	0	6
Luwero	0	10	3	1	14	10	0	0.0350	7
Kassanda	5	6	2	0	13	10	0	0.0699	7
Total	12	38	8	3	61	41			

Key: HC-Health Center; HF-Health Facility, DR-Decision Rule

In each district, we sampled the HFs using simple random sampling without replacement.

### Sampling of Villages in the HF Catchment Areas

Within each HF catchment area, we sampled 6 villages/interview locations using simple random sampling with replacement after weighting the villages for population. In the villages, we interviewed mothers or caregivers of CwDs aged 0-59 months old living in the HF's catchment area. For HF-based data or records, we similarly sampled 6 clients or 6 data points from the relevant sources. This rule excludes assessment for inputs like human resources or equipment for which we assessed or observed only one data point in the HF. The sample size of 6 respondents per HF has previously been employed in LQAS r-HFA surveys based on 95% “pU”, 50% “pL”, maximum tolerable α and β of 0.11 each. These parameters yield a DR of 5 and actual α and β errors of 0.0328 and 0.1094 respectively^20^. The DR of 5 means that a HF is classified to have acceptable performance in a given indicator if at least 5 of the 6 sampled respondents or data points have the correct response (i.e., the outcome of interest).

For the community-based data, a list of villages in the HF catchment area was compiled. Because the HF in-charges did not have the actual population of the villages, they were asked to rank the villages based on population; a village was given a rank of 3 if it had a large population, a rank of 2 if it had a moderate population, and a rank of 1 if it had a small population in comparison to the others in the catchment area. Given the HF in-charges' experience in the area, they were able to know villages with many or few people although they lack actual numbers. Following that, the list of villages was rewritten, with a village being written the number of times its population was ranked in order to meet the probability proportional to size, with villages with larger populations having a higher chance of being chosen than villages with smaller populations. For example, once a village had been ranked as highly populated and awarded three marks, it was written three times in the village list. Then simple random sampling was employed with replacement to select six villages where the interviews would be conducted in the community. These sampling procedures produced 246 respondents from the 41 HFs in the catchment (project) area.

To select the appropriate respondents in the village, segmentation sampling was employed. A village map was drawn in the selected village, segmented and a segment randomly sampled at every point the segments had an approximately equal number of households. The village was sub-divided, segment randomly sampled until the randomly sampled segment had 15 or fewer households that are considered to be manageable as is recommended in the guidelines.^21,22,23^,^24^ At this point, the list of households in the final segment was generated from which a reference household was randomly sampled. The eligible respondent was then looked for from the household nearest to the front door of the reference household. This procedure was done until the number of respondents needed in the village was obtained after which the data collector proceeded to the next sampled village until all 6 interviews in the HF catchment area were obtained.

### Indicators

Sixteen indicators were used to assess service coverage along the constructs of: provision of child disability-related services to CwDs, access to rehabilitation services, readiness of the HFs to provide basic disability management and rehabilitation services, existence of community structures for linkage to disability management and rehabilitation services, psychosocial service provision, and promotion of the human rights of CwDs.

### Data Collection

Data on the presence of at least one health worker and at least five VHT members who had ever been trained in basic child disability management and rehabilitation service provision were collected at the HFs. The remaining indicators were investigated through a community survey, but the results reflected the HF's performance in providing CDR services within its catchment area. The trained data collectors interviewed mothers or caregivers of CwD in the HF catchment areas. Only one mother or caregiver was interviewed in a sampled interview location although some villages were sampled more than once. For example, if a village was sampled twice, two interviews were conducted. A new random starting point was determined for the second interview in a village that was chosen more than once.

### Data Analysis

The coverage of the CDR services was obtained at the HF, district, and catchment area (project) level. At the HF level, for a district to have performed satisfactorily on a particular indicator, at least 5 of the 6 respondents or data points must have the correct response (i.e., the outcome of interest).^25^ The number of HFs with satisfactory performance was added for each district and compared against the district's DR for each indicator. Any district that had HFs with satisfactory performance equal to or surpassing the DR had attained the 80% coverage target and its performance was adjudged satisfactory for the reference indicator. The number of HFs with satisfactory performance were aggregated for the entire catchment area, i.e., the four districts and the project-level proportion (percent) of HFs with satisfactory performance was established in our second level of analysis. The obtained percentage coverage was compared against the 80% performance target. Any indicator with a coverage target of 80% or more had satisfactory overall performance or otherwise if less than 80%. The district-specific coverage estimates for the population-level data/indicators were also calculated (Table 3) but were not the focus of the study as our major focus was centred on the performance of the HFs.

### Quality Assurance

Two members of the study team who are also experts in conducting LQAS-based surveys reviewed the tools and protocol. The research assistants were trained for three days, with one day spent pre-testing the tools in Kajjansi Health Center IV and its catchment area in Wakiso district. To ensure coherent understanding when asking questions to mothers/respondents who did not speak English, the tools were translated into the local language (Luganda) that is spoken by majority of people in the study area. The data collection process was overseen by three of the authors (SPK, JM, and MS). Daily debriefs were held to ensure that the data was of high quality.

### Ethical Approval

This study received ethical approval from the Mildmay Uganda Research Ethics Committee and the National Council of Science and Technology, with approval numbers REC REF0603-2020 and HS896ES respectively. Permission to conduct the study in each district and at each HF was obtained from the District Directors of Health Services (DHO) and HF in-charges. Written informed consent was obtained from the parents or caregivers of CwDs prior to conducting interviews. Parents of children chosen for community survey participation were physically asked for written informed consent. The parents who refused to participate in the study were replaced.

The parents and caregivers of CwD were provided with sufficient information about the risks and benefits of their children participating in this study, as well as consent and confidentiality concerns. Parents were informed of their options for withdrawing from the study after having consent. The importance of confidentiality was emphasized during the training, particularly with regard to patient records and respondent information. The names of the participants in the study were not written on any of the data collection tools or mentioned in the report.

## RESULTS

There were 60 respondents each from Kassanda and Luwero, 78 from Mityana, and 48 from Mubende. The majority of respondents (60.1%) were caregivers of CwD aged 23-59 months. Most of the respondents had children with physical disabilities. Table 2 reveals the characteristics of the respondents.

**Table 2.** Characteristics of Children with Disabilities Aged 0 to 59 Months

Variable	Frequency	Percent
District		
Kassanda	60	24.4
Luwero	60	24.4
Mityana	78	31.7
Mubende	48	19.5
Total	246	100
Age category		
0-11months	85	34.7
12-23 months	13	5.2
23-59 months	148	60.1
Total	246	100
The types of disabilities the CwD in the study had		
Physical disability	81	32.9
Multiple disabilities	55	22.4
Mental retardation	21	8.5
Visual impairment	17	6.9
Speech or language impairment	16	6.5
Hearing impairment	15	6.1
Hearing and visual impairment	5	2.0
Emotional disturbance	2	0.8
Others	32	13.0
Total	246	100

**Table 3.** Population Coverage Estimates for Child Disability Services

Construct Indicator	Mubende (n=48)	Kassanda (n=60)	Mityana (n=78)	Luwero (n=60)	Row Percentage
**Care of CwDs**					
CwDs seen by a trained health care provider on an issue related to ther disability during the last 1 year	28 (58.3%; 95% CI: ±13.9)	39 (65%; 95% CI: ±12.1)	55 (70.5%; 95% CI: ±10.1)	36 (60%; 95% CI: ±12.4)	158 (64.2%; 95% CI: ±6)
CwDs who received all for age required child health services	7 (14.6% 95% CI: ±10)	12 (20%; 95% CI: ±10.1)	15 (19.2%; 95% CI: ±8.7)	12 (%; 95% CI: ±10.1)	46 (18.7%; 95% CI: ±4.9)
**Access to rehabilitation services**					
CwDs receiving rehabilitation services with evidence of active engagement in care	11 (22.9%; 95% CI: ±11.9)	20 (33.3%; 95% CI: ±11.9)	33 (42.3%; 95% CI: ±11)	25 (41.7%; 95% CI: ±12.5)	89 (36.2%; 95% CI: ±12.2)
**Psychosocial care to CwDs**					
Caregivers or parents of CwDs who acknowledge receiving any socioeconomic support from a person or an organization or being part of social protection programme of persons with disabilities	10 (20.8%; 95% CI: ±11.5)	7 (11.7%; 95% CI: ± 8.1)	6 (7.7%; 95% CI: ±5.9)	6 (10%; 95% CI: ±7.6)	29 (11.8%; 95% CI: ±4)
Caregivers of CwDs who acknowledge that a household member got trained and sensitized on offering support for persons with disabilities	10 (20.8 %; 95% CI: ±11.5)	15 (25%; 95% CI: ±11)	16 (20.5 %; 95% CI: ±9)	6 (10%; 95% CI: ±7.6)	47 (19.1%; 95% CI: ±4.9)
Caregivers of CwDs who acknowledge currently engaging in any business to raise money to cater for a CwD and other children	23 (47.9%; 95% CI: ±14.1)	14 (23.3%; 95% CI: ±10.7)	16 (20.5 %; 95% CI: ±9)	8 (13.3 %; 95% CI: ±8.6)	61 (24.8%; 95% CI: ±5.4)
Caregivers of CwDs who acknowledge having received any support from family members to help a CwD	8 (16.7%; 95% CI: ±10.6)	13 (21.7%; 95% CI: ±10.4)	14 (17.9%; 95% CI: ± 8.5)	5 (8.3%; 95% CI: ±7)	40 (16.3%; 95% CI: ±4.6)
Caregivers of CwDs that report having received any support for people with a disability from community persons or any organization	6 (12.5 %; 95% CI: ±9.4)	7 (11.7 %; 95% CI: ±8.1)	10 (12.8%; 95% CI: ±7.4)	1 (1.7%; 95% CI: ±2.9)	24 (9.8%; 95% CI: ±3.7)
**Respect of human rights for CwDs within communities and families**					
Caregivers of CwDs who accept that when they visit the HF for health services, health workers give special attention to their children with a disability	45 (93.8%; 95% CI: ±6.8)	57 (95%; 95% CI: ±5.5)	76 (97.4%; 95% CI: ±3.5)	56 (93.3%; 95% CI: ±6.3)	234 (95.1%; 95% CI: ± 2.7)
Caregivers of CwDs who stated that their children never experienced any form of violence from a family member	41 (85.4%; 95% CI: ±10)	54 (90%; 95% CI: ±7.6)	73 (93.6%; 95% CI: ±5.6)	57 (95%; 95% CI: ±5.5)	225 (91.5%; 95% CI: ±3.5)
Parents or caregivers of CwDs who report that that a child never experienced any form of violence from a community member	41 (85.4 %; 95% CI: ±10)	59 (98.3%; 95% CI: ±2.9)	74 (94.9%; 95% CI: ±4.9)	59 (98.3 %; 95% CI: ±2.9)	233 (94.7%; 95% CI: ±2.8)
Caregivers of CwDs report that they sought for justice in the courts of law, local council or any organization that offers help to CwDs for a child who faced violence from a family member (n=21)	*	*	*	*	2 (10.0%; 95% CI: ±22.8)**
Caregivers of CwDs who report that they sought for justice in the courts of law, local council or any organization that offers help to CwDs for a child who faced violence from a community member (n=13)	*	*	*	*	4 (30.7%; 95% CI: ±26.2)**

* Calculation of coverage not possible due to very low sample size

** Large CI. The denominator was made of respondents who reported that their CwD faced violence (from family or community member)

### Population coverage estimates for child disability services

Table 3 presents the results of the population coverage estimates for services delivery to CwDs in the respective districts and the unweighted coverage for the project area.

### Population coverage estimates for care of CwDs

#### Visiting a health worker for disability related services

During the year prior to the evaluation, 64.2% (95% CI: 58.2-70.2) of CwDs consulted a trained healthcare provider for disability-related concerns. Conversely, only 18.7% (95% CI: 13.8-23.6) of CwDs received all age-appropriate health services, including immunizations, Vitamin A supplementation, deworming, and slept under mosquito nets to prevent malaria.

Regarding rehabilitation services, only 36.2% (95% CI: 24.0-48.4) of CwDs were undergoing rehabilitation. Solely 11.8% (95% CI: 7.8-15.8) of caregivers received socioeconomic support or participated in disability social protection programs. Only 19.1% (95% CI: 14.2-24.0) of caregivers reported family members being trained to support CwDs, while 16.3% (95% CI: 11.7-20.9) acknowledged family assistance. Additionally, 24.8% (95% CI: 19.4-30.2) of caregivers engaged in business to support their disabled child. Coverage for caregivers who received community or organizational disability aid was 9.8% (95% CI: 6.1-13.5).

Community and family respect for CwDs was notably high, with 95.1% (95% CI: 92.4-97.8) of caregivers affirming special attention from healthcare workers during HF visits. Additionally, 91.5% (95% CI: 88.0-95.0) of caregivers of CwDs stated that their children had never faced violence within the family, while 94.7% (95% CI: 91.9-97.5) reported no violence from community members. Yet, when such abuse occurred, few sought justice. For instance, only 10% (2 out of 21) of caregivers whose children experienced family abuse pursued legal recourse, and 30.8% (4 out of 13) sought justice for community abuses. Despite high coverage in respect indicators, achieving justice for CwDs remains a challenge when they face abuse from family or community members.

### The LQAS based DRs and HF coverage of child disability services

In this section, we present the LQAS HF coverage estimates and DRs for classifying districts as performing acceptably well or poorly in terms of disability services access and delivery in their catchment areas, as well as attainment or non-attainment of the DRs set. Table 4 shows the results of the r-HFA on service coverage for CwDs in the four districts, as well as the DRs reached.

**Table 4.** LQAS-Classification of Districts' Coverage in Core Indicators for CDR Services

Construct/Indicator	MBD (n=8) DR=5	KSD (n=10) DR=6	MTN (n=13) DR=8	LWR (n=10) DR=6	Percent	95% CI
**Care of CwDs**						
HFs in which at least 5 of the 6 sampled CwDs was seen by a trained health care provider for the disability during the past 1 year	2	5	6	4	41.5%	±15.1
HFs in which at least 5 of the 6 sampled CwDs received all-for-age required child health services	0	1	2	2	12.2%	±10
**Use of rehabilitation services**						
HFs in which at least 5 of the 6 sampled CwDs are receiving rehabilitation services with evidence of active engagement in care	0	1	1	3	12.2%	±10
**Readiness of the HFs to provide basic CDR services**						
^b^HFs with at least one health worker trained in disability detection, management and rehabilitation in the last two years	5a	4	12a	10a	75.6%	±14.2
^b^HFs that have basic rehabilitation toolsφ to offer disability management care	5a	4	12a	7a	68.3%	±14.4
**Community structures for CDR linkage**						
^b^Villages in HF catchment area having a VHT trained in disability detection, management and rehabilitation	5a	6a	6	5	53.7%	±15.3
**Psychosocial care to CwDs**						
HFs in which at least 5 of the sampled 6 caregivers or parents of CwDs acknowledge receiving any socioeconomic support from a person or an organization or being part of social protection program of persons with disabilities	0	0	0	0	0	0
HFs in which at least 5 of the sampled 6 caregivers of CwDs acknowledge that a household member got trained and sensitized on offering support for persons with disabilities	1	0	0	0	2.4%	±4.7
HFs in which at least 5 of the sampled 6 caregivers of a CwDs acknowledge currently engaging in any business to raise money to cater for CwDs and other children	1	0	0	0	2.4%	±4.7
HFs in which at least 5 of the sampled 6 caregivers of CwDs acknowledge having received any support from family members to help CwDs	0	0	1	0	2.4%	±4.7
HFs in which at least 5 of the sampled 6 caregivers of CwDs report having received any support for people with a disability from community members or an organization	0	0	0	0	0	0
**Respect for rights of CwDs within communities and families**						
HFs in which at least 5 of the 6 sampled caregivers of CwDs report that health workers give special attention to their CwDs	6a	9a	13a	9a	90.2%	±9.1
HFs in which at least 5 of the 6 sampled caregivers of CwDs report that their children never experienced any form of violence from a family member during the past 12 months	6a	9a	12a	9a	87.8%	±10.3
HFs in which at least 5 of the 6 sampled caregivers of CwDs report that the CwD never experienced any form of violence from a community member during the past 12 months	5a	10a	12a	10a	90.4%	±7.3

a District met the DR based on the 80% coverage target

b The indicator coverage is based on one data point

c The HF was required to have a wheelchair, walkers, play material, play mattresses, a paediatric coach and a rustication kit

Note: MBD = Mubende district; KSD = Kassanda district; MTN = Mityana district; and LWR = Luwero district

#### Care of children with disabilities

HFs were assessed if they adequately provide care for CwDs. Only 41.5% (95% CI: 26.4-56.6) of the HFs had at least 5 of the 6 CwDs seen by trained healthcare providers for disability-related issues during the 12 months preceding the study – none of the districts attained the DR. The evaluation also included assessments of whether the CwDs adequately accessed routine prevention services like scheduled vaccinations, maternal nutrition education, deworming tablets, vitamin A administration, and long-lasting insecticide-treated mosquito net use. It was expected that each child receives all the services. Only 12.2% (95% CI: 2.2-22.2) of HFs had at least 5 of the 6 sampled children adequately receive age-appropriate child-disability prevention services, and all the districts fell short of the DR.

#### Readiness of the HFs to provide basic CDR services

The r-HFA assessed HFs' readiness to offer critical child disability detection, management, and rehabilitation services. This necessitated the presence of essential tools like wheelchairs, walkers, play materials, mattresses, a pediatric coach, and a rustication kit. Only Kassanda district fell short of the DR regarding number of its HFs that had the basic rehabilitation tools/equipment. Overall, 68.3% (95% CI: 53.9-82.7) of HFs possessed these tools, and this falls below the 80% threshold for adequate coverage. The coverage of HFs with at least one health worker trained in child disability prevention and management in the past two years stood at 75.6% (95% CI: 61.4-89.8), slightly below the 80% target. Kassanda was the only district falling short of the DR.

### Existence of community Structures for Child Disability Rehabilitation Linkage

Assessment of availability of community structures included HFs having VHTs who are offering community-based CDR services like caregiver counseling and linking CwDs to care. Luwero and Mityana districts fell short of the DR, and 53.7% (95% CI: 38.4-69.0) of HFs had VHTs to provide community-based CDR services.

#### Use of Rehabilitation Services

We found that only 12.2% (95% CI: 2.2-22.2) of HFs had at least 5 of the 6 sampled CwDs were actively receive rehabilitation services that are appropriate for their age.

#### Coverage of the Psychosocial Care to CwDs and their Caregivers

In enhancing the rehabilitation process, alongside health services provision, emphasis was placed on bolstering the psychosocial support for caregivers of CwDs. However, this aspect showed poor coverage and performance; none of the HFs had at least 5 out of the 6 sampled mothers receive socioeconomic support or participate in social protection programs for persons with disabilities. Similarly, only 2.4% (95% CI: -2.3-7.1) of HFs had at least 5 of the 6 caregivers of CwD receive family support. No district attained the DR in anyone of the indicators used to measure coverage extend of psychosocial support. Comparable (low) coverage was observed for HFs where at least 5 of the 6 caregivers affirm household members' training for CwD support. Additionally, HFs in which adequate coverage of caregivers engaged in business for CwDs' welfare and those receiving community or organizational disability support had similar low coverage levels (Table 4).

### Respect for Rights of Children with Disabilities Within Communities and Families

Safeguarding the rights of CwDs showed varied coverage across indicators. Notably, 90.2% (95% CI: 81.1-99.3) of HFs had at least 5 out of the 6 sampled caregivers acknowledge special attention from health workers for CwDs. Additionally, 87.8% (95% CI: 77.5-98.1) of the HFs had at least 5 out of the 6 caregivers state that the CwDs in their care did not experience violence perpetrated by a family member, and 90.4% (95% CI: 83.1-97.7) of them reported that no member of the community perpetrated violence towards CwDs in the past year. Despite instances of reported violence, justice for the affected CwDs remained elusive. Only 2 out of 10 caregivers sought justice for CwDs facing community violence, while none pursued legal recourse for violence inflicted by family members.

## DISCUSSION

The HF assessment, combined with a community survey, provides an account of the CDR project's coverage, which included the provision of disability detection, management, referral, and rehabilitation services to CwDs after two years of intervention provision. Our findings show poor care for CwDs; only 41.5% and 12.2% of the HFs had satisfactory performance in terms of CwDs being seen by a trained provider and receiving all-for-age required child health services during the one year preceding the survey, respectively, with no district-level variation because all districts fell short of the predetermined performance target.

These findings suggest that many CwDs had not visited a HF to receive appropriate care or healthcare advice, indicating access challenges for CwDs not only for rehabilitative services but also for routine healthcare. Only 12.2% of the HFs had satisfactory use of rehabilitative services, indicating a low level of current active engagement in rehabilitative care by CwDs. This observation is not unique to our study; previous studies have reported difficulties with access to services for people with disabilities in LMICs ^26 27 28^. Barriers include stigmatization, distance to facilities, financial constraints, infrastructure issues, and negative attitudes from healthcare personnel.^27,29^ In this HFs' study context, child-disability care was a recent addition to routine services. Advocacy efforts within communities were limited, and those benefitting failed to spread awareness effectively leading to low awareness and utilization among caregivers.^14^ Besides, services were dependent on implementing partners' resource availability and this, coupled with challenges in geographical access may have further compounded the provision of the services. The findings call for addressing barriers to access which is crucial for improving service delivery.

A notable proportion of HFs demonstrated preparedness to offer services for CwDs. Our survey found that 68.2% of the HFs were equipped with essential rehabilitative tools. Although overall performance fell short of the 80% target, this, more-than-expected (high) percentage of HFs with essential tools indicates room for improvement.^30^ District-level analysis uncovered variations in performance, with Mubende, Mityana, and Luwero districts exhibiting better preparedness compared to Kassanda, suggesting potential areas for targeted interventions and resource allocation to address disparities and enhance service delivery. The findings highlight a paradox that despite a sizeable number of HFs being well suited to offer child disability services, these children did not utilize the services to satisfaction. This suggests potential barriers or gaps in access, awareness, or other factors hindering their uptake. Thus, understanding such factors leading to low utilization informs resource allocation, service improvement strategies, and policy development. If such barriers are addressed, this is likely to enhance service impact, benefitting CwDs' health outcomes and quality of life.

The findings also reveal that over 75.6% of the HFs had trained health workers in child disability detection, management and rehabilitation, and 53.7% of them had trained VHTs as community-level structure to offer community-based services and connect the CwDs to advanced care. It is encouraging to see that more than 75.6% of HFs had their health workers trained in preventing, managing and rehabilitating child disability, which aligns with literature highlighting the crucial role of trained professionals in enhancing the lives of CwDs.^31^ Training of health workers enlightens them on the various types of child disabilities and provides them with better information on referral pathways for rehabilitation services based on the children's disabilities.^32^ The training is also necessary to change health workers' perceptions of CwDs and any implicit or explicit stigma they may have towards these children.^29^ This could explain why, according to 90.2% of CwDs' caregivers, whenever CwDs visited HFs for any services, health workers paid special attention to them. Furthermore, the fact that 53.7% of HFs had trained VHTs emphasizes the significance of community-based services and social support in improving access to health services as revealed in other settings.^33,34^ It is thus necessary to increase HFs with such structures to improve the community-level rehabilitation services and linkages to care for CwDs.

The findings also reveal that caregivers of CwDs performed poorly in terms of psychosocial services and economic empowerment. The project's services included forming caregiver support groups, encouraging family members and relatives to support CwDs, and educating caregivers on how to support their children. Others included forming savings groups to help caregivers improve their financial situation in order to empower them to care for CwDs. It has been shown that when psychosocial services for caregivers of CwDs are strong and functional, parents and caregivers are more likely to take their children for disability care.^9^ As a result of receiving psychosocial support from their peers in support groups, the caregivers become more empowered to support their children. Counseling provided to caregivers during their meetings also contributes to the reduction of the social stigma associated with disability. However, the insufficient psychosocial assistance for caregivers of CwDs as seen in this study meant persistence in increased stress, mental health problems, a reduced quality of life, family instability, restricted access to resources, and detrimental effects on the child's development.^35^ When caregivers fail to receive psychosocial services, the CwDs negatively get affected since they are less likely to be given a dignified life by their caretakers. This is exacerbated by their relatives' near-inadequate assistance as well as a lack of community, civil society, and government support. The importance of having family members and relatives participate in the care of CwDs in order to foster a conducive relationship for CwDs' growth has been emphasized.^36^ These situations necessitate improved strategies to ensure that the intentions of psychosocial and economic support are realized.

The evaluation of whether the human rights of CwDs are maintained yielded mixed outcomes. Health workers offering special attention to CwDs when they visited HFs as well as family and community members upholding children's rights showed satisfactory coverage of above 80% in all districts. However, caregivers seeking justice for violated rights of CwDs exhibited poor outcomes. The mixed results seen in the evaluation findings demonstrate the complexity of CwD-related human rights issues. The notion that health workers offer special attention to CwDs and family and community members support their rights is consistent with the idea that CwDs are entitled to fundamental rights, including the right to dignified healthcare.^37^ The United Nations Convention on the Rights of the Child emphasizes the importance of protecting the rights of all children, including CwDs.^38^ The failure to seek justice for violated CwDs is a violation of these rights. Failure to seek justice for violated CwDs jeopardizes their path to a dignified life while also causing psychological torture to mothers who are already struggling to care for the child.^34^ The situation is exacerbated if family members are involved in obstructing the pursuit of justice for CwDs whose rights have been violated. This could be worrying in a situation like the study districts where a few family members were giving minimal support to caregivers of CwDs. This complexity underscores the importance of upholding CwDs' rights and addressing barriers to justice-seeking.

There are some limitations to this study that should be noted. First, there was no baseline study of which the results could have been used to compare the effectiveness of the interventions. As a result, attributions for satisfactory and unsatisfactory coverage are difficult. While the project interventions were carried out in a few communities within the catchment areas of the sampled HF, all communities within the HF's catchment area were considered, which may have resulted in an underestimation of project intervention coverage. Data on violence against disabled children by a family member was self-reported. This may result in reporting bias, especially if a child had ever been violated and the respondent concealed this information. If the respondent is violent towards the CwD, it is possible that they did not provide accurate information.

## CONCLUSION

Despite the Community Disability Rehabilitation project's efforts, the findings of this study revealed inadequate care for CwDs, with barriers to access, awareness, and limited resources still contributing to low CwDs service utilization. However, the basic rehabilitation tools provided at the HFs although not adequate, and the health workers who had been trained to offer these services act as starting points for potential improvement. Addressing the gaps seen in this study is critical for enhancing services and health outcomes. Some positives are evident at the outcome level, such as a high-level observation of the rights of CwDs at HFs, family, and community levels, despite few attempts to seek legal redress in cases of rights violations. A critical and structured analysis of service delivery bottlenecks and an examination of their possible root causes are necessary to develop evidence-based strategies for holistic improvement in the delivery and uptake of child disability prevention, management, and rehabilitation across the continuum.
